# Impact of a postpartum care rehabilitation program to prevent postpartum depression at a secondary municipal hospital in Qingdao China: a cross-sectional study

**DOI:** 10.1186/s12884-023-05547-z

**Published:** 2023-04-11

**Authors:** Xiaoqian Zhang, Xiaoli Zuo, Catharina Matheï, Bert Aertgeerts, Masoud Afnan, Tang Li, Frank Buntinx, Mieke Vermandere

**Affiliations:** 1 Academic Center for General Practice, Department of Public Health and Primary Care, Leuven, KU Belgium; 2 Qingdao United Family Hospital, Qingdao, China; 3 Qingdao University Medical College, Qingdao, China

**Keywords:** Postpartum rehabilitation, Postpartum practices, Postpartum depression, China, Doing the month

## Abstract

**Background:**

The emerging postpartum rehabilitation (PPR) program in Chinese hospitals characterized by applying ongoing medical care through traditional cultural practices shows a protective effect in early puerperium in China. This study explores the benefit of PPR program practices to postpartum depression (PPD) and the influencing factors for PPD among Chinese women during the first postnatal six weeks.

**Methods:**

The cross-sectional study included 403 participants and was conducted in a Secondary Municipal Hospital in Qingdao, China, from 01 to 2018 to 31 December 2021. Information on this PPR program was collected during the six-weeks postpartum consultation, including the Edinburgh postnatal depression scale (EPDS) scores, the measurement results for diastasis recti abdominis, and the international physical activity questionnaire (long form) (IPAQ-L) scores. Logistic regression models were used to examine the effect of the PPR program on PPD among the local population. The secondary aim of this study was to investigate possible influencing factors for PPD, such as coronavirus disease 2019 (COVID-19), physical exercises, etc.

**Results:**

PPR program has shown a positive effect in preventing PPD (p < 0.001) and diastasis recti prevalence (p < 0.001) during the six-weeks postnatal control in Qingdao, China. Better post-pregnancy weight reduction (p = 0.04) and higher metabolic equivalent of task (MET) value (p < 0.001) were noticed in the non-PPR group. Furthermore, lower PPD risk was associated with factors such as longer relationship duration years (2–5 years) (p = 0.04) and exercising one to three times a week (p = 0.01). A higher PPD risk was related to factors such as urinary incontinence during the postpartum period (p = 0.04) and subjective insomnia (p < 0.001). No significant effect was shown between COVID-19 and the EPDS score in this study (p = 0.50).

**Conclusion:**

Our results suggested that the PPR program provided protection against PPD and diastasis recti during the first six weeks after delivery. Urinary incontinence and subjective insomnia were the main risk factors for PPD, while longer relationship duration years and exercising one to three times a week gave protective effects to PPD. This study emphasized that a comprehensive ongoing medical care program, such as the PPR program, effectively improves women’s mental and physical health in the early postpartum in China.

**Supplementary Information:**

The online version contains supplementary material available at 10.1186/s12884-023-05547-z.

## Background

The Chinese traditional confinement ‘doing the month’ or ‘zuo yue zi’ [1, 2], refers to a number of cultural practices and health beliefs that influence some Chinese women and east Asian women. These practices include strict prescriptions and proscriptions, food taboos, the use of traditional herbs, avoiding outdoor activities, refraining from washing, etc. [1, 3]. According to some women who advocate these traditions, following these rituals is important for recovering and preventing future illness, regardless of whether these rituals are supported by scientific evidence or not [4]. Conceptually, the majority of Chinese women still adhere to these traditional beliefs and practices [4, 5].Furthermore, due to the quality of life improvements, more and more maternal care centers have emerged in the recent decade, mostly in first-tier cities in China [6]. These are professional maternal health care facilities operated by registered companies or hospitals for taking care of both mothers and their babies, enabling the practice of ‘doing the month’ to be carried out [7]. Women who decide to stay in such care centers pay more attention to their postpartum recovery quality [8]. Studies have shown that an extended postpartum hospital stay assists women to recuperate better, both physically and emotionally [9, 10]. In order to improve the quality of postnatal care for these mothers, medical professionals need to be more aware of how to modify the postpartum care to fit the unique culture of ‘doing the month’ [4].

Recently, a novel service model has been established in certain Chinese hospitals for individuals who want to adhere to the tradition of ‘doing the month’ while simultaneously receiving professional postpartum care [11]. Multi-disciplinary health professionals like family physicians, pediatricians, obstetricians, traditional Chinese medical doctors, nutritionists, midwifes, breastfeeding instructors, nurses, physical therapists, patient servicers, and other co-workers collaborated in this specially designed postpartum care program to provide comprehensive medical care to help both mothers and babies. Necessary physical therapies, routine ward rounding by physicians with different specialties, nursing cares, and other services such as various exercise classes are provided during the hospital stay (see supplementary file 1). The purpose of this postpartum rehabilitation program (PPR) is to manage the delivered women’s health in a more evidence-based manner during ‘doing the month’ period. However, little information is available in the literature to determine whether these maternal centers would benefit the health of women after delivery [8, 12]. Some studies have shown that a high-quality ‘doing the month’ experience positively impacted maternal parenting quality [4, 14–16] and exercises can be effective in reducing the symptoms of postpartum depression [14, 17], while some restrictions of ‘doing the month’ practices might negatively impact the mental health of postpartum women [18, 19]. We hypothesized that women who joined such PPR program would have a lower postpartum depression (PPD) risk on week six after delivery. The main aim of this study was to examine the effect of the new PPR program to PPD among the local Chinese women. Additionally, limited studies have shown a negative effect of the coronavirus disease 2019 (COVID-19) to PPD [20, 21] and a beneficial effect from undertaking physical activities during the puerperium [22]. A secondary aim of this study was to investigate whether the PPD risk correlates with COVID-19, physical exercises, and other complications.

## Methods

### Study design and sample

This cross-sectional study was conducted at United Family Hospital (UFH) in Qingdao, China using consecutive sampling, between December 2018 and December 2021. The researchers consecutively invited all patients who attended their six-week postpartum follow-up consultations at Qingdao UFH on a fix pre-scheduled workday every week to participate in the study. After obtaining written informed consent, the participant was asked to complete questionnaires with the assistance of the interviewer. The inclusion criteria for the participants were as follows: (1) patients aged 18 and older (2) Ambulatory outpatients who were able to visit the clinic unassisted (3) Patients coming for their six-week postpartum visit (4) Patients from whom written informed consent was obtained (5) Patients were prepared to complete a self-reported complications questionnaire and receive predefined physical examinations (6) Ability to understand Chinese or English language. Women with a history of mental health disorders, chronic pain, severe diseases that make it difficult to come for the additional ambulatory follow up visits, severe pregnancy complications (preeclampsia/eclampsia, placenta previa, placental abruption, major birth defects, low birth weight (< 1500 gram [23]), still birth, handicap, and women who do not understand Chinese or English were excluded. All participants were guided by trained researchers to complete the questionnaires after a planned consultation in the obstetric department. The power calculation was conducted using online open software OpenEpi [24]. Our sample size was calculated based on the mean and standard deviation of the EPDS score as reported in a previous study as well as the estimates obtained in our pretest stage [25]. The estimated sample size was 129 subjects in each group (total 258 participants), with 80% power and 95% confidence interval.

As the trained researchers were full-time medical staff members who volunteered their spare time to assist with this study, and as a result of covid regulations, we attempted to collect the participants’ information on a set workday during the week. Additional survey questions concerning postpartum practices, demographic status, and complications were reviewed by experts and pretested prior to being collected.

### Data collection

Participants were recruited from 01 to 2018 to 31 December 2021 during their six-week postpartum checkup in the Qingdao UFH PPR center by using consecutive sampling method. Participants were introduced to a research doctor after getting their verbal permission when their planned routine check-up appointment was complete. After introduction to the trained co-worker, an information sheet and written informed consent form were given to the patient. After the participant signed the informed consent, interviews, physical examinations, and questionnaires were completed through a face-to-face interview with the patient by trained researcher in a physically separated room. Specifically, the patient completed demographic information, a post-partum depression questionnaire, a physical activity questionnaire and was examined for diastasis recti. Participants were divided into two groups based on whether they have ever participated in the PPR program during their first month following delivery. It is intended that the information of this study was not shared with participants at the moment of delivery, so that they could make their own decision regarding whether to enroll in the PPR program or not. The final outcomes (results of the questionnaires and patient’s personal information data) were not shared with the treating physician or any other medical staff in the hospital. The signed consent form and patient’s identification information were both kept confidentially by the main researcher (ZXQ) with one hard copy to ensure the patient’s privacy.

### Socio-demographic and clinical measures

Participants were asked to provide their age, height, delivery method, educational level, family income, marital or relationship duration, occupation status, newborn gender, the expectation of the newborn gender, parity numbers, and information regarding planned or unplanned pregnancy.

### Assessment of postpartum depression symptoms

Postpartum depression was measured with the validated Chinese version of the Edinburgh postnatal depression scale (EPDS) [26–28], aimed to indicate increasing symptoms, explored mood, pleasure, guilt, anxiety, fear, ability to cope, insomnia, sadness, and self-injury [29]. Each question on the EPDS is graded on a 4-point scale (range 0–3), resulting in a total score that ranges from 0 to 30, with scores of 10 or more suggesting potential PPD. The cutoff 9/10 was chosen instead of the threshold 12/13 in English version [28, 30] to achieve a better sensitivity [26].

### Assessment of physical exercises

The physical activity was measured through a validated Chinese version of the International Physical Activity Questionnaire (long form) (IPAQ-L) [31–33], and the data from the questionnaire were transformed into energy expenditure estimates as metabolic equivalents (METs) in hours per week of activity. The method to calculate “minutes per week” is shown in the formula as shown below (Table 1). The total physical activity of postpartum women was categorized as low (< 600 MET·min·wk-1), moderate (600 ~ 3000 MET·min·wk-1), or high (> 3000 MET·min·wk-1) physical activity [34], corresponding to less than 150 min per week, 150 ~ 750 min per week, or more than 750 min per week of moderate physical activity. This self-administered, long form of IPAQ contains four domains of physical activity: work-related, transportation, housework or gardening and leisure-time activity. The questionnaire also includes data about time spent sitting as an indicator of sedentary behavior.

**Table 1:** MET value for different physical activities

Domain	MET value
Work	Moderate MET value = 4.0 Vigorous MET value = 8.0
Transport	Cycling and walking MET value = 4.0
Recreation	Moderate MET value = 4.0 Vigorous MET value = 8.0

Minute per week = 0.825 × walking minutes + 1 × moderate minutes + 1.375 × garden minutes + 1.5 × cycling minutes + 2 × vigorous minutes

### Assessment of diastasis recti

Diastasis recti abdominis (DRA) is a medical condition in which the rectus abdominis muscles are separated by an abnormal distance without any fascia defect [35]. DRA could be measured by palpating 4.5 cm above, at, and 4.5 cm below the umbilicus in a standardized supine crook-lying position with both arms crossed over the chest [36]. Patients were asked to perform an abdominal crunch till the shoulder blades were off the bench. DRA will be categorized into five categories as non-DRA (separation < 2 fingerbreadths), mild DRA (from 2 fingerbreadths to less than 3 fingerbreadths), moderate DRA (from 3 fingerbreadths to less than 4 fingerbreadths) and severe DRA ( ≧ 4 fingerbreadths). Observed protrusion along the linea alba was categorized as DRA With Protrusion, even if the palpated distance was less than 2 fingerbreadths [36].

### Statistical analysis

The data analyses were conducted with the Statistical Package for the Social Science (SPSS for Windows 14.0, SPSS Inc., Chicago, IL). Potential confounders including socio-demographic variables, primary caregivers, exercise times per week, complications during the postpartum period, etc. were reported (Table 2). In addition, we adjusted for parity, expected gender of the newborn baby, and feeding mode to evaluate the influence of these factors. EPDS values, IPAQ data and diastasis recti value were recorded as means with standard deviations. Univariate analysis followed by a multivariate logistic regression analysis were used to calculate the odds ratio (ORs) for different risk factors. Final significance was set at P < 0.05 and Ors with the 95% confidence intervals (95% CI) were considered significant. There was no missing data.

**Table 2:** The socio-demographic and obstetric characteristics of the PPR and non-PPR groups

Variables (n = 403)	Non-PPR (n = 146)	PPR (n = 257)	Total N(%)	ORu (95%CI)	p
Age (Mean ± SD)	30.84 ± 4.12	31.67 ± 3.83			
< 30 year-old	53	77	130(32.3)	1	
≥ 30–34 year-old	68	126	194(48.1)	1.49 (0.83,2.68)	0.19
≥ 35 year-old	25	54	79(19.6)	1.16(0.67,2.04)	0.59
Height (Mean ± SD)	165.37 ± 5.07	165.20 ± 5.29			
≤ 165 cm	79	130	209(51.8)	1	
> 165 cm	67	127	194(48.1)	1.15(0.77,1.73)	0.50
Delivery method					
C/S	30	64	94(23.3)	1	
NVD	116	193	309(76.7)	0.78(0.48,1.27)	0.32
Pelvic floor assessment					
Normal	18	44	62(15.38)	1	
Hypertonic pelvic floor	2	12	14(3.47)	0.65(0.33,1.29)	0.22
Hypotonic pelvic floor	89	142	231(57.32)	0.27(0.06,1.26)	0.09
Mixed type of pelvic floor	37	59	96(23.82)	1.00(0.61,1.63)	1.00
Urinary incontinence before pregnancy					
Yes	10	19	29(7.2)	1	
no	136	238	374(92.8)	0.92(0.42,2.04)	0.84
Income of the core family members					
< 8000 RMB per month	37	52	89(22.1)	1	
> 8000 RMB per month	109	205	314(77.9)	1.34(0.83,2.17)	0.24
Education level of the participant					
Lower than college diploma	5	5	10(2.5)	1	
College and above	141	252	393(97.5)	1.79(0.51,6.28)	0.36
Occupation of the participant					
White collar	50	93	143(35.5)	1	
Self-employed	69	129	198(49.1)	0.70(0.38,1.28)	0.25
Housewife/others	27	35	62(15.4)	0.69(0.39,1.24)	0.22
Planned pregnancy					
Yes	69	144	213(52.9)	1	
No	77	113	190(47.1)	0.70(0.47,1.06)	0.09
Newborn gender					
Male	78	129	207(51.4)	1	
Female	68	128	196(48.6)	1.14(0.76,1.71)	0.53
Expressed wish for baby gender					
None	100	173	273(67.7)	1	
Wish for a girl	27	52	79(19.6)	0.97(0.52,1.81)	0.93
Wish for a boy	19	32	51(12.7)	0.87(0.42,1.82)	0.72
Marriage or relationship length duration (Mean ± SD)	3.81 ± 2.99	4.42 ± 3.73			
≤ 2 years	62	103	165(40.9)	1	
> 2, < 5 years	36	66	136(33.7)	1.10(0.69,1.77)	0.68
≥ 5 years	48	88	102(25.3)	1.00(0.58,1.71)	1.00
Parity					
Primipara	95	177	272(67.5)	1	
Multipara	51	80	131(32.5)	0.84(0.55,1.30)	0.43

OR: the odds ratio of univariate logistic regression analysis, with 95% confidence intervals

N = 403 (98.3% of 410) after exclusion of missing data for all covariates in multivariate analysis

SD: Standard deviation

### Ethical approval

The procedures were approved by the Qingdao Affiliated University of Qingdao [37], Qingdao United Family hospital, and agreed by the Medical Ethical Board of the KU Leuven (S62625). All participants completed the written informed consent before the study after verbal briefing by the researcher.

### Main findings

#### **Participants**

As previously mentioned, participants were assessed on a fixed pre-scheduled workday of the week, due to availability of the researcher and COVID regulations. From December 2018 to December 2021, 410 women were invited to participate in this study. A total of 403 valid questionnaires were collected with an effective participation rate of 98.3% (Seven refusals). The non-PPR group consisted of 146 participants while the PPR group consisted of 257 participants. All of the including participants signed the consent forms and completed the questionnaires fully. The age range was between 20 to 45-year-old. There were no significant differences in the socio-demographic and obstetric characteristics between the PPR and non-PPR groups (Table 2).

### Main findings

#### **PPR and postpartum depression**

Patients who participated in the PPR program had a lower EPDS score (p < 0.001) using logistic regression (Table 3). In total, 66 (16.4%) women were scored as high risk for PPD (≥ 10) on the EPDS, with 35 in non-PPR group and 31 in PPR group. The non-PPR group had a clearly higher average EPDS score than the PPR group (6.25 vs. 4.64, ORu = 2.30, 95% CI = 1.35–3.92) (ORu = univariate odds ratios).

**Table 3:** Univariate and multivariate logistic regression model for related factors for the PPR and non-PPR group

Variables	Non-PPR (n = 146)	PPR (n = 257)	ORu (95%CI)	p	ORm (95%CI)	p
EPDS score (Mean ± SD)	6.25 ± 5.27	4.64 ± 4.14				
≤ 9	111	226	1		1	1
≥ 10	35	31	2.30(1.35,3.92)	0.00	2.35(1.36,4.07)	0.00
Diastasis recti						
separation < 2 fingerbreadths	135	250	1		1	1
separation ≥ 2 fingerbreadths	11	7	2.91(1.10,7.68)	0.03	3.62(1.36,9.65)	0.01
MET value						
< 600 MET·min·wk-1	102	203	1		1	1
600 ~ 3000 MET·min·wk-1	27	48	0.18(0.07,0.46)	0.00	0.18(0.07,0.46)	0.00
> 3000 MET·min·wk-1	17	6	0.20(0.07,0.56)	0.00	0.19(0.07,0.54)	0.00
Weight differences between post and pre-pregnancy (post – pre pregnancy weight)						
< 5 kg	60	133	1		1	1
≥ 5 kg	86	124	0.65(0.43,0.98)	0.04	NS	0.08
Main care giver to the newborn						
Parents	73	122	1		1	1
Grandparents or other family members	15	13	1.26(0.82,1.93)	0.29	NS	0.84
Nanny or babysitter from maternal center	58	122	2.43(1.08,5.43)	0.03	NS	0.07
Type of feeding						
100% breastfeeding	88	185	1			
> 50% breastfeeding	37	42	0.48(0.17,1.31)	0.15	-	-
< 50% breastfeeding	13	22	0.88(0.30,2.58)	0.82	-	-
100% bottle feeding	8	8	0.59(0.18,1.95)	0.39	-	-
Sleep time						
< 7 h per night	44	70	1		-	-
7–8 h per night	71	131	1.14(0.64,2.03)	0.67	-	-
> 8 h per night	31	56	0.98(0.58,1.66)	0.94	-	-
Exercise frequency						
Less than once weekly	68	126	1			
Once weekly	15	29	0.93(0.56,1.54)	0.77	-	-
2–3 times weekly	28	42	0.89(0.42,1.88)	0.75	-	-
> 3 times weekly	35	60	1.14(0.61,2.16)	0.68	-	-
Complications during postpartum period Breast problem						
No	76	122	1		-	-
Yes	70	135	1.20(0.80,1.80)	0.38	-	-
Wound problem						
No	137	227	1		-	-
Yes	9	30	2.01(0.93,4.36)	0.08	-	-
Pain						
No	108	179	1		-	-
Yes	38	78	1.24(0.79,1.95)	0.36	-	-
Insomnia						
No	121	198	1		-	-
Yes	25	59	1.44(0.86,2.43)	0.17	-	-
Constipation						
No	111	186	1		-	-
Yes	35	71	1.21(0.76,1.93)	0.42	-	-
Urinary incontinence						
No	132	237	1		-	-
Yes	14	20	0.53(0.39,1.63)	0.53	-	-
Hemorrhoids						
No	100	192	1		-	-
Yes	46	65	0.74(0.47,1.15)	0.74	-	-
Pelvic organs prolapse						
No	144	253	1		-	-
Yes	2	4	1.14(0.21,6.29)	0.88	-	-

*NS* not significant

##### **PPR and diastasis recti**

A better diastasis recti recovery rate (ORu = 2.91, 95% CI = 1.10–7.68) was also found in the PPR group (Table 3).

##### **PPR and physical exercise**

The MET value was significantly higher in the non PPR group, shown in both univariate and multivariate regression models (Table 3).

##### **PPR and other postnatal complications**

None of the eight specified complications (urinary incontinence / wound / breasts / pain / sleep / constipation / hemorrhoids / pelvic organ prolapse problems) showed any difference between the two groups (Table 3).

Although a greater post-pregnancy weight reduction was noticed in the non-PPR group on univariate regression analysis, this result was not significant in the multivariate regression model (Table 3).

##### **Other influencing factors for PPD**

In addition to the PPR program we analyzed the data for associations between other factors and PPD. The new coronavirus COVID-19 was transmitted between human beings in China since January 2020. This has declared the outbreak as a pandemic in March 2020 (WHO) [38]. Univariate logistic regression analysis was conducted to evaluate factors, such as the COVID-19, physical exercises, and other complications on the postpartum depression risk. No significant correlation was shown in this study between the COVID-19, the MET value itself, and the diastasis recti prevalence rate with the PPD risk. A lower PPD risk was associated with exercising one to three times a week (p = 0.01, ORu = 0.37, 95% CI = 0.17–0.79), and longer marriage or relationship duration years (2–5 years) (p = 0.04, ORu = 0.51, 95% CI = 0.26–0.96) (Table 4). Meanwhile, a higher PPD risk was associated with urinary incontinence during postpartum period (p = 0.04, ORu = 2.33, 95% CI = 1.06–5.14) and subjective insomnia (p < 0.001, ORu = 3.13, 95% CI = 1.77–5.52), but not related with the total sleeping time (Table 4).

**Table 4:** Comparison of the possible influencing factors of the different EPDS groups (≤ 9 and ≥ 10)

Variables	EPDS ≤ 9 (n = 337) n/%	EPDS ≥ 10 (n = 66) n/%	ORu	Beta coefficient	*p*
Diastasis recti					
separation < 2 fingerbreadths	17(5.04)	1(1.52)	1	1	
separation ≥ 2 fingerbreadths	320(94.96)	65(98.48)	3.45(0.45,26.41)	1.24	0.23
MET Value					
< 600 MET·min·wk-1	258(76.56)	47(71.21)	1	1	
600 ~ 3000 MET·min·wk-1	62(18.40)	13(19.70)	1.94(0.73,5.17)	0.66	0.19
> 3000 MET·min·wk-1	17(5.04)	6(9.09)	1.68(0.56,5.09)	0.52	0.36
Marriage or relationship duration					
≤ 2 years	129(38.28)	36(54.55)	1	1	
> 2, < 5 years	89(26.41)	13(19.70)	0.51(0.27,0.96)	-0.76	0.04
≥ 5 years	119(35.31)	17(25.76)	0.98(0.45,2.12)	-0.22	0.96
Exercises times/week					
<once per week	151(44.81)	43(65.15)	1	1	
1–3 times weekly	100(29.61)	14(21.21)	0.37(0.17,0.79)	-1.00	0.01
> 3 times weekly	86(25.52)	9(13.64)	0.75(0.31,1.81)	-0.29	0.52
PPR/Non-PPR					
PPR group	226(67.06)	31(46.97)	1	1	
Non-PPR group	111(32.94)	35(53.03)	2.30(1.35,3.92)	0.83	0.00
Having planned pregnancy					
Yes	183(54.30)	30(45.45)	1	1	
No	154(45.70)	36(54.55)	1.43(0.84,2.42)	0.36	0.19
Insomnia					
No	279(82.79)	40(60.61)	1	1	
Yes	58(17.21)	26(39.39)	3.13(1.77,5.53)	1.14	0.00
Sleeping time					
< 7 h per night	92(27.30)	22(33.33)	1	1	
7–8 h per night	169(50.15)	33(50.00)	0.61(0.28,1.33)	-0.50	0.21
> 8 h per night	76(22.55)	11(16.67)	0.74(0.36,1.54)	-0.30	0.42
Urinary incontinence					
No Yes	313(92.9) 24(7.1)	56(84.8) 10(15.2)	1 2.33(1.06,5.14)	1 0.85	0.04
COVID					
Before	113(33.5)	25(37.9)	1	1	
After	224(66.5)	41(62.1)	0.83(0.48,1.43)	-0.19	0.50

ORu: the odds ratio of univariate logistic regression analysis, with 95% confidence intervals

## Discussion

### Main results

The findings of our study provide evidence of a positive impact of a hospital-based postpartum rehabilitation (PPR) program on postpartum depression in the first 6 weeks following delivery.

The proportion of women with a high risk of PPD (EPDS score of ≥ 10) was 12.0% in the PPR group compared with 23.9% in the non-PPR group (p < 0.001). As can be seen in other studies, good environmental factors such as healthier lifestyle-related factors, appropriate social support, and better interpersonal relationships are associated with better postpartum mental health [39, 40]. Interestingly, the PPR program also had a significant positive effect on the reduction of the prevalence of diastasis recti. This is likely due to the fact that the PPR group received extra individual specified physical trainings during the first four weeks after delivery, led by experienced physical therapists. This finding is consistent with previous studies that specified post-partum physical exercises may be effective for reducing the prevalence of diastasis recti [41, 42].

### PPR and physical exercise

This study found that both the dose of physical activity (MET value) and the post-pregnancy weight reduction over the six weeks were higher in the non-PPR group. Although the PPR group received extra sport exercises during their month-long stay, other social activities were very limited due to the fact that they were served with daily foods, nanny services and received medical attention on site. The need for outdoor activities therefore became unnecessary for these people. The non-PPR group had more opportunities for outdoor activities, such as buying life supplies and maintaining social contacts. This may explain why the PPR women had fewer daily activities compared with the non-PPR group after discharge. Focusing on encouraging a sport habit rather than giving passive sport lessons during the first month may be more beneficial for new mothers’ weight management and PPD risk control in the future.

### Additional influencing factors for postpartum depression

#### COVID-19

Contrary to other research, although the outdoor activities were very limited to the new mothers, the prevalence of PPD was not significantly higher during the COVID-19 period in this study. We consider the following reasons to explain this phenomenon in our study: First, parents / parents-in-law may use their own experiences and parenting methods to influence the new mothers and the babies in China. When the ideas of parenting are different from both parties, it may cause family conflicts, frustrations, and negative emotions to new mothers [43]. During the COVID period, parents visiting was very limited and even forbidden. This would give the new fathers more chance to take care of the babies and strengthen the relationship between husbands and wives. Secondly, China’s “dynamic zero-COVID” policy has resulted in very few positive cases being reported in Qingdao in the past two years compared to other countries [44, 45]. The negative influence on psychological health by lockdown restrictions during the COVID-19 epidemic to the local residents was not significantly related with the PPD risk in this study.

#### Physical exercise

In this research endeavour, an investigation was conducted to analyze the relationship between the dose and frequency of physical activity and PPD. Our results indicate that engaging in physical activity one to three times per week was associated with a decreased risk of PPD. However, we found no discernible correlation between PPD and the dose of physical activity, as measured by MET value. Overall, our study findings provide support for the theory that physical activity during the postpartum period could potentially diminish the prevalence of PPD. This outcome may be attributed to the emotional impact of exercise, which can enhance self-confidence and alleviate negative thoughts [46]. Other studies have also illustrated the positive impact of physical activity on the risk of PPD [15]. Further exploration, particularly through subgroup analysis, would be beneficial in determining whether the effects were a consequence of specific dose or frequency levels of physical activity.

#### Other complications

##### **Relationship duration**

The study showed a lower risk of PPD when the marriage or relationship duration of the couples was between 2 and 5 years. These findings indicated that having a baby very shortly (< 2 years) after marriage was a potential risk factor for PPD. This phenomenon might be related with the Chinese social system, which didn’t benefit new mothers for their career promotion path. Having a baby shortly after marriage might create more challenges to the new mother, including complicated family members’ relationships and different socioeconomic status. On the other hand, the risk of PPD increased when the marriage or relationship duration was more than 5 years. Marital satisfaction has strong negative correlation with the prevalence of PPD, which in turn was generally negatively predicted by the duration of marriage [37, 47, 48]. This risk factor for PPD should be studied in the future to improve the mothers’ mental health.

##### **Subjective insomnia**

An interesting finding in this study was that the average amount of sleeping hours was not related to PPD, but a subjective experience of insomnia does. While women with an increased risk for PPD may sleep the same number of hours as other delivered mothers, they may feel as if they are not getting enough sleep. The expectation of getting a certain number of sleeping hours or the subjective feeling of sleeplessness may be related with PPD. Breastfeeding mothers need to breastfeed the babies every two to four hours during the first few months causing an interrupted night’s rest. The extra stress created by an interrupted sleep pattern can negatively affect the patient’s mood. How the new mothers cope with this stress might affect their mental state rather than the absolute sleeping hours. More studies on how this experience of insomnia affect the PPD would be useful in the future.

##### **Urinary incontinence**

We also found that urinary incontinence had a significant correlation with the PPD. One of the reasons might be that this complication was often perceived as a stigma in traditional Chinese culture. This negative attitudes towards urinary incontinence inhibits patients from seeking care. Additional attention should be spent on these complications by medical staff, actively asking and checking the patient in a comfortable and private environment.

### Strength and limitations

This study has investigated the advantage of a pre-existing PPR program in Qingdao, China. It delivered additional information about the risk factors of PPD, partly during the COVID-19 pandemic. The strength of this study is the direct comparison of two groups in a real-world setting. However, several limitations should be considered. First of all, an intervention study comparing two or more randomized comparison groups is more suited to determine the causal relationship than the cross-sectional study design. Yet, such design would not be possible against the background of Chinese customs. Second, our study group was only collected in a secondary municipal hospital in an urban area. This study was conducted in Qingdao, the second biggest city of Shandong province. Our population has a significant higher family income than the average income of other communities. Finally, this study is not double-blinded that the researchers knew the group information of the participants prior to the interviews.

### Implications for practice and future research

Our PPR program has more medical input than most PPR programs in China. It would be interesting to investigate other new existing postpartum care programs’ clinical efficacy, including cost-benefit, cost-effectiveness, and cost-utility analysis.

We found associations between duration of the relationship or marriage, urinary incontinence and the feeling of sleeplessness and PPD. These should all be studied further. We also found that despite the patient receiving exercise interventions during the “month”, that this pattern of behavior did not carry through beyond the month. Given the known beneficial effect of exercise [49, 50] this area would benefit from further study.

Finally, we did not see any significant association of the COVID-19 pandemic and PPD. This was different from other studies, possibly due to other factors, such as the environment [21, 51, 52]. Future studies may explore such relationships.

## Conclusion

This study has shown a strong association between the PPR program and a reduction in prevalence of PPD symptoms and diastasis recti during the first six weeks after delivery. Urinary incontinence and subjective insomnia were the main risk factors for PPD, while relationship duration between 2 and 5 years and exercising one to three times a week gave protective effects to PPD. Based on the findings of this study, it is evident that a comprehensive program of ongoing care, specifically the PPR program, is crucial for promoting the physical and mental well-being of women during the early postpartum period in China. These results carry significant policy implications for the development and organization of postpartum care services in the country.

## Electronic supplementary material

Below is the link to the electronic supplementary material.

**Additional file 1: Supplementary File 1.** Services for postpartum women during ‘doing the month’ period in different settings in Qingdao

## Data Availability

The authors are happy to share anonymized data related to this paper upon receiving a specific request, along with the purpose of that request. Interested parties may contact 2187zhang@gmail.com.

## Abbreviations

PPR: Postpartum rehabilitation
PPD: Postpartum depression
COVID-19: coronavirus disease 2019
EPDS: Edinburgh Postnatal Depression Scale
UFH: United Family Hospital
IPAQ-L: International Physical Activity Questionnaire – Long Form
DRA: Diastasis Recti Abdominis
WHO: World health organization
MET: Metabolic equivalent of task
95% CIs: 95% confidence intervals
SD: Standard deviation
ORs: Odds ratios
