# Supplementary material for: Impact of a postpartum care rehabilitation program to prevent postpartum depression at a secondary municipal hospital in Qingdao China: a cross-sectional study
Source: BMC Pregnancy Childbirth. 2023 Apr 11;23:239. doi: 10.1186/s12884-023-05547-z (PMC10088113; doi:10.1186/s12884-023-05547-z)
Supplement: Supplementary file 1 — Additional file 1: Supplementary File 1. Services for postpartum women during ‘doing the month’ period in different settings in Qingdao [file 12884_2023_5547_MOESM1_ESM.docx]

| **Different services** | **Frequency of services for traditional ‘doing the month’ at home** | **Frequency of services in a Traditional Chinese Postpartum care center** | **Frequency of services in Qingdao United family hospital postpartum rehabilitation department** |
| --- | --- | --- | --- |
| **Physical therapy** | None | Mostly none | 5 times per week |
| **Medical services gynecologists** | None | Mostly once weekly | 3 times per week |
| **Medical services pediatricians** | None | Mostly once per week | 3 times per week in the first 2 weeks, once per week from third week |
| **Nutritionist consultation** | None | Mostly once per week | Once per week |
| **Traditional Chinese Medicine doctor consultation** | None | Mostly once per week | Once per week |
| **Family medicine doctor consultation** | None | None | Once per week |
| **Baby massage & Baby bubble bath** | None | Mostly none or once per week | After umbilical cord separation, 1 to 4 times per week. |
| **Nursing care** | None | Mostly 24h, 7 days per week available | 24 h, 7 days per week available |
| **Urgent care** | None | None | 24 h, 7 days per week available |
| **Group exercise session** | Dependent on  different individuals’ lifestyle | Mostly none | 2 times per week |

**Supplementary File 1: Services for postpartum women during ‘doing the month’ period in different settings in Qingdao**
